# The impact of gratitude interventions on patients with cardiovascular disease: a systematic review

**DOI:** 10.3389/fpsyg.2023.1243598

**Published:** 2023-09-21

**Authors:** Xiaoxiao Wang, Chunli Song

**Affiliations:** The Second Hospital affiliated to Jilin University, Changchun, Jilin, China

**Keywords:** systematic review, cardiovascular disease, gratitude, positive psychology, mechanism

## Abstract

Positive psychological factors play a pivotal role in improving cardiovascular outcomes. Gratitude interventions are among the most effective positive psychological interventions, with potential clinical applications in cardiology practice. To better understand the potential clinical effects of gratitude interventions in cardiovascular disease, four databases (Web of Science, Scopus, PubMed, and PsycArticles) were searched from 2005 to 2023 for relevant studies. Randomized controlled trials of gratitude interventions as the intervention and that reported physiological or psychosocial outcomes were eligible for inclusion. In total, 19 studies were identified, reporting results from 2951 participants from 19 to 71 years old from both healthy populations and those with clinical diagnoses. The studies showed that gratitude not only promotes mental health and adherence to healthy behaviors but also improves cardiovascular outcomes. Gratitude may have a positive impact on biomarkers of cardiovascular disease risk, especially asymptomatic heart failure, cardiovascular function, and autonomic nervous system activity.

## 1. Introduction

Cardiovascular disease (CVD) is a complex disease with a multifactorial etiology and a persistent, recurrent, and severe clinical course that can result in serious consequences for the patient’s quality of life (QoL). Consequently, both patients and clinicians find it difficult to manage these conditions. As a result, psychological interventions are particularly important in patients with CVD.

Within preventive cardiology and positive psychology, targets to improve patients’ mental health are established to reduce the risk of CVD and increase QoL (Labarthe et al., 2016). Good mental health includes the presence of positive psychological factors such as happiness (Akosile et al., 2018), optimism (Tindle et al., 2009; Huffman et al., 2016a; Weiss-Faratci et al., 2017), emotional vitality (Kubzansky, 2007), sense of purpose (Kim et al., 2013, 2019), life satisfaction (Lopuszanska et al., 2013; Sun et al., 2022) and mindfulness (Levine et al., 2017), which are associated with decreased risk and outcomes of CVD. Negative mental health is also multifaceted and may be characterized by chronic stress (Richardson et al., 2012; Steptoe and Kivimaki, 2013), anger (Chida and Steptoe, 2009; Mostofsky et al., 2014), anxiety (Emdin et al., 2016), depression (Gan et al., 2014) and pessimism (Pankalainen et al., 2016), which can negatively affect cardiovascular health. Studies should be performed investigating mental health as an independent predictor of cardiovascular health (Kubzansky et al., 2018). The growth of “gratitude research” in recent years has provided a new perspective on psychological interventions for patients with CVD. Gratitude (a topic of growing interest in positive psychology research) focuses on an individual’s potentially beneficial sentiments and converts them into a positive healing force. It has been found that gratitude therapy is independently related to lowering the risk of CVD (Cousin et al., 2021). Furthermore, gratitude therapy is considered beneficial in slowing the progression and improving the prognosis of CVD (Mills et al., 2015; Jackowska et al., 2016; Millstein et al., 2016; Redwine et al., 2016; Huffman et al., 2016a). However, there is currently insufficient knowledge regarding the potential benefits of gratitude therapy among healthcare workers (Cousin et al., 2021). Hence, in this review, we discuss the current status of overseas and domestic research on the effects of gratitude in the cardiovascular field. We aimed to shed a new light on gratitude intervention research and provide a reference for future clinical application.

## 2. Search strategy and inclusion criteria

**Figure 1:**
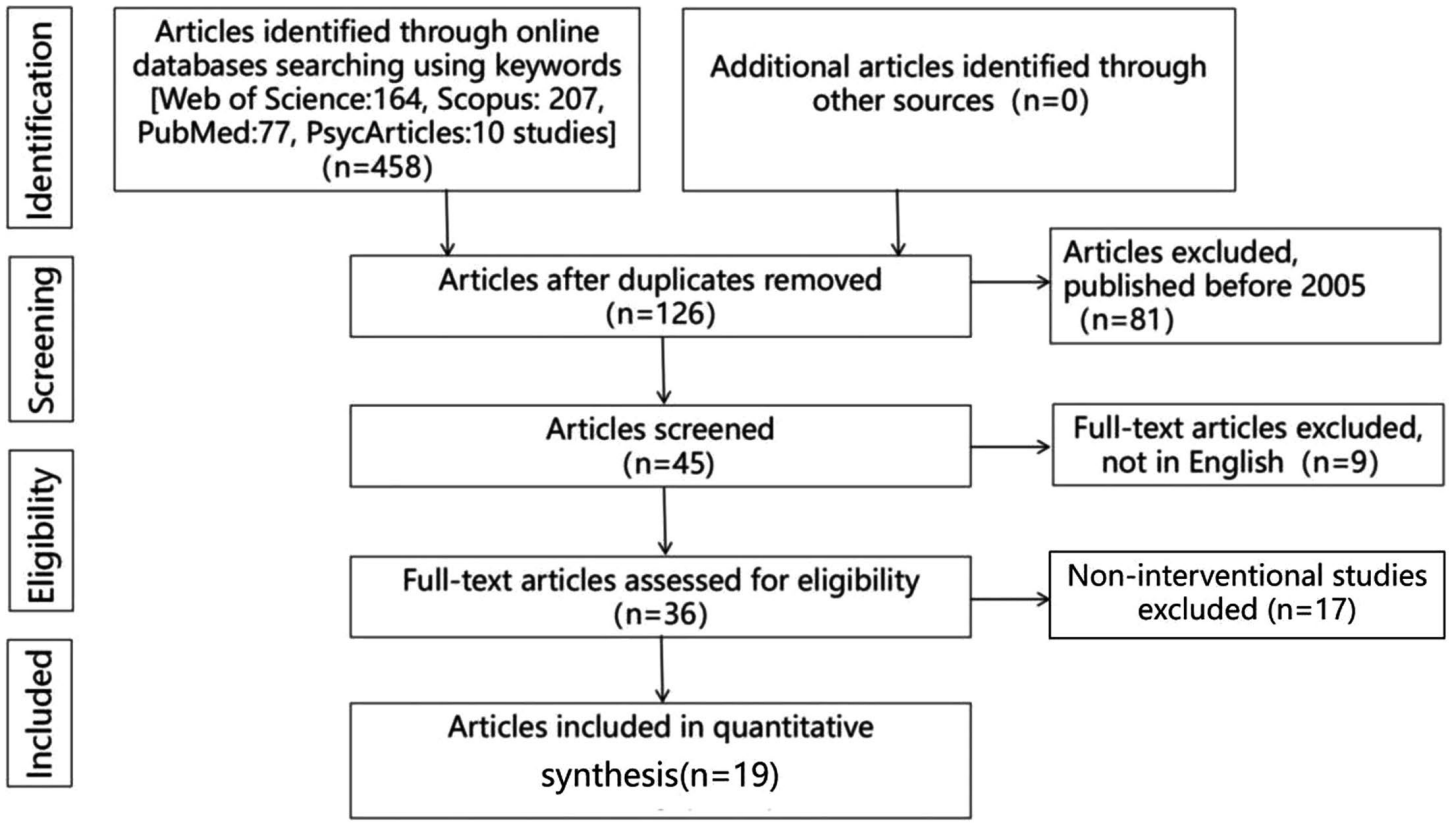
Diagram of selecting articles for the systematic review.

We searched major databases including Web of Science, Scopus, PubMed, and PsycArticles for recently published studies, limited to the years 2005 to 2023, using keywords related to the topic of gratitude among patients with cardiovascular disease. More specifically, we used keywords such as “gratitude,” “thanksgiving,” “cardiovascular disease,” “cardiovascular diseases,” “cardiology,” “heart failure,” “acute coronary syndromes,” and “coronary heart disease” to retrieve full-text, quantitative articles in English. This systematic review adhered to the PRISMA guidelines. Finally, 19 published articles were evaluated in this systematic review. The process is summarized in Figure 1. The included articles (*n* = 19) were thoroughly read, and the titles and main findings are summarized in Table 1.

**Table 1:** Summary of articles related to gratitude studies.

First author (year)	Study design	Type participant	*N*	Mean Age (SD)	% Male	Gratitude measure(s)	Intervention	Time frame	Summary of findings
Redwine et al. (2016)	Randomized blinded trial	Patients with stage B heart failure	70	66(7.58)	95%	GQ6	Gratitude journal	8 weeks	Gratitude was associated with lower levels of inflammatory biomarkers, such as CRP, TNF-α, IL-6 and sTNFr1, and higher parasympathetic heart rate variability.
Moieni et al. (2019)	Randomized blinded trial	Healthy middle-aged women	76	43(4.70)	0%	GQ6	Writing aimed at inducing gratitude	6 weeks	Gratitude interventions had no direct effect on inflammatory markers. Gratitude interventions were correlated with a decreased percentage of monocytes producing proinflammatory IL-6 and TNF- α through the habit of increased support-giving.
Schache et al. (2020)	Randomized blinded trial	Type 1 diabetes patients	80	NR	NR	GQ6	Gratitude journal	8 weeks	Gratitude interventions can help improve glucose control in patients with type 1 diabetes.
Wolfe and Patterson (2017)	Randomized blinded trial	Undergraduates	140	20 (6.93)	0%	–	Gratitude listing	2 weeks	Gratitude interventions were demonstrated to effectively reduce eating disorders, lower the risk of depressive symptoms and reduce negative psychological outcomes.
Fritz et al. (2019)	Randomized blinded trial	Undergraduates (1) and high school students (2)	327(1) 1017(2)	(1):19(1.30) (2):NR	24%(1) NR (2)	GQ6	Gratitude letter	(1):2 weeks (2):4 weeks	Gratitude interventions can improve eating behaviors.
Sultan et al. (2018)	Randomized blinded trial	Patients with CHD	40	48.64	100%	GQ6	An intervention that focuses on enhancing positive psychological attributes	8 weeks	Higher levels of gratitude and lower CHD symptoms were observed in the positive psychological intervention group. Positive psychology interventions can facilitate gratitude and help reduce the risk of CHD.
Jackowska et al. (2016)	Randomized blinded trial	Young women	119	26	0%	–	Gratitude journal	2 weeks	Gratitude interventions are associated with a decrease in diastolic blood pressure and improvements in depression and sleep quality when compared with the control.
Digdon and Koble, (2011)	Randomized blinded trial	Undergraduates	41	23 (6.11)	22%	–	Positive events journaling	1 week	Improvements in sleep quality and quantity and reductions in the level of pre-sleep arousal were observed after the gratitude intervention.
O’Connell et al. (2017)	Randomized blinded trial	Mainly young adult sample	192	27(12.60)	33%	GQ6	Reflective behavior – reflective only – control journaling	3 times a week for 3 weeks	Gratitude interventions had great potential in decreasing negative feelings and reducing their adverse effects on health.
Salces-Cubero et al. (2019)	Randomized blinded trial	Elderly	124	69(7.78)	40%	–	Positive activities available to train gratitude	One week before intervention, 1 week after intervention, and a month after intervention completion	Gratitude interventions were associated with decreases in negative mental states and increases in positive psychological states, which can enhance patients’ subjective well-being and life satisfaction.
Yang et al. (2018)	Randomized blinded trial	Prisoners	144	NR	NR	–	Gratitude journal and group seminars	6 weeks	Gratitude interventions can decrease negative mental states and significantly enhance the subjective well-being of prisoners.
Cheng et al. (2015)	Randomized blinded trial	Medical workers	102	NR	35%	–	Gratitude journal	Twice a week for 4 weeks	Gratitude interventions were correlated with decreased self-reported depression symptoms and perceived stress.
Ramírez et al. (2014)	Randomized blinded trial	Elderly	46	71 (7.06)	65%	–	Gratitude letter	Once a week for 9 weeks	Gratitude interventions can significantly reduce depressive symptoms and anxiety and enhance subjective well-being and life satisfaction.
Watkins et al. (2015)	Randomized blinded trial	College students	129	NR	29%	SGRAT	Gratitude journal	6 weeks	Gratitude interventions can reduce depressive symptoms and enhance subjective happiness over the course of 5 weeks after the intervention.
Southwell and Gould, (2017)	Randomized blinded trial	Persons with depression or anxiety disorders	109	34(10.80)	12%	GQ6	Gratitude journal	3 weeks	Participants with a high level of gratitude have lower scores on sleep difficulties, anxiety and depression.
Hazlett et al. (2021)	Randomized blinded trial	Healthy women	61	43(4.80)	0%	–	Writing aimed at inducing gratitude	Once a week for 6 weeks	Gratitude interventions may decrease sympathetic nervous system activity by reducing amygdala reactivity and activating the ventral striatum and diaphragm area.
Kyeong et al. (2017)	Randomized blinded trial	Healthy people	32	23 (2.50)	47%	–	Gratitude and resentment interventions	5 min	The average heart rate during the gratitude intervention was lower than the average heart rate during the resentment intervention
Rash et al. (2011)	Randomized blinded trial	Adult sample	56	23 (3.00)	54%	GQ6	Gratitude contemplation	Twice a week for 4 weeks	Gratitude practices can reduce stress, increase cardiac coherence and result in more ordered ECG waveforms and greater physiological coordination.
Matvienko-Sikar and Dockray, (2017)	Randomized blinded trial	Pregnant women	46	34(3.04)	0%	GDP	Gratitude journal	Four times a week for 3 weeks	The gratitude intervention group showed lower cortisol and stress levels in wakefulness and sleep during pregnancy compared to the control group that received conventional therapy.

ACS, acute coronary syndrome; CHD, coronary heart disease; ET-1, endothelin-1; GDP, the Gratitude during Pregnancy Scale; GQ6, gratitude questionnaire 6; GRAT, gratitude, resentment, and appreciation test; NR, not reported; SGRAT, short gratitude, resentment, and appreciation test; sICAM-1, soluble intercellular adhesion molecule-1.

## 3. What is gratitude?

Seligman and Csikszentmihalyi (2014) proposed the concept of positive psychology, applying interventions to increase positive feelings, at the end of the 20th century. With the rise of positive psychology, gratitude has gradually become an important research topic (Guo et al., 2022). Gratitude refers to the psychological tendency to understand or respond the help of others with appreciative emotions and behaviors (McCullough et al., 2002). Gratitude is a mental orientation toward the appreciation of others and life circumstances (Wood et al., 2010) and a positive affective state experienced when people receive help (McCullough et al., 2002). In a clinical sense, gratitude can inspire clinical workers to change their perspective by augmenting their ability to find appreciation, allow them to assist patients to transform it into a positive force and help individuals cope with stress effectively. To improve cardiovascular health, gratitude may also be a positive psychological asset to have. Furthermore, gratitude can be divided into a trait and state level. State level refers to a positive emotion experienced when an individual receives external favors, whereas the dispositional trait level refers to a general orientation toward perceiving and appreciating the positives in life (Wood et al., 2010; Leung and Tong, 2017). People who have a high trait gratitude tend to experience and express it more easily, frequently, and strongly.

## 4. Relationship between gratitude and cardiovascular disease

Gratitude impacts CVD in many ways. For patients with CVD, gratitude may be effective in relieving somatic symptoms and increasing regular functioning of the cardiovascular system, including inflammation control. It also helps to promote healthy behaviors and reduce the potential for sleep disorders (Boggiss et al., 2020).

### 4.1. Inflammation

In a randomized blinded trial, Redwine et al. (2016) found that circulating inflammatory markers were reduced in patients with asymptomatic heart failure (HF) after an 8-week gratitude intervention. Furthermore, a study by Mills et al. (2015) found that gratitude was associated with reduced inflammatory biomarker levels, including CRP, TNF-α, IL-6, IFN-γ, and ST2. Redwine et al. (2016) and Hartanto et al. (2019) found that decreases in personality gratitude were significantly associated with decreases in IL-6 in 1054 adults. For Moieni et al. (2019), while a direct effect on inflammatory markers was not observed, it was concluded that the effect of gratitude on inflammation is an extremely complex process. It is highly recommended that further large-scale experimental investigations into other biological indicators, including inflammatory indices, be performed before drawing conclusions.

### 4.2. Health behaviors and factors

The American Heart Association proposed 7 parameters regarding healthy lifestyle behaviors (smoking, diet, physical activity, body weight) and metabolic measures (plasma glucose, cholesterol, blood pressure) to reduce the risk of CVD and promote optimal cardiovascular health (Lloyd-Jones et al., 2010). Hypertension, hypercholesterolemia, and cigarette smoking are well-known and modifiable risk factors for various CVD largely influenced by behavioral routines. It is estimated that 80% of CVDs are considered preventable through engagement in healthy behaviors (Masters et al., 2020). Gratitude may facilitate the establishment of health-promoting behaviors in individuals, such as medication compliance (Cousin et al., 2020), appropriate glucose/lipid control, and healthy eating (Wolfe and Patterson, 2017; Fritz et al., 2019; Schache et al., 2020). A study by Redwine et al. (2016) found that gratitude interventions can increase medication adherence and delay the progression of heart failure by improving self-efficacy. In a randomized blinded trial, Redwine et al. (2016) and Fritz et al. (2019) found that gratitude interventions can improve eating behaviors. Furthermore, another randomized blinded trial by Redwine et al. (2016) and Wolfe and Patterson (2017) found that gratitude interventions were demonstrated to effectively reduce eating disorders and lower the risk of depressive symptoms. A randomized blinded trial by Redwine et al. (2016) and Schache et al. (2020) found that gratitude interventions can help improve glucose control in patients with type 1 diabetes. In a study involving 1775 adults, Krause et al. (2017) reported that increases in gratitude were correlated to improvements in hemoglobin A1c levels. Moreover, recent studies have revealed that gratitude interventions after acute coronary syndromes (ACS) are associated with elevated adherence to health behaviors that influence cardiometabolic health, including diet, physical activity, and medication adherence (Millstein et al., 2016). Additionally, several studies have suggested that gratitude may impact the occurrence and development of CVD by increasing a wide variety of health behaviors and positive health outcomes (Sacco et al., 2014; Huffman et al., 2016b; Sultan et al., 2018; Legler et al., 2019).

### 4.3. Sleep

Furthermore, gratitude can reduce the risk of CVD by improving sleep quality. In a single-blind randomized controlled trial among young females, Jackowska et al. (2016) found that gratitude interventions improved the quality and duration of sleep and boosted energy recovery when compared with the control. In a randomized blinded trial among undergraduates, improvements in sleep quality and quantity and reductions in the level of pre-sleep arousal were observed after writing gratitude journals (Digdon and Koble, 2011). A study by Mills et al. (2015) and Redwine et al. (2016) found that gratitude was associated with improvements in depression and sleep quality among patients with stage B HF. It is critical to change pre-sleep cognitive processes, including anxiety, fear of sleep or nightmares, and rumination, that potentially interfere with the processes that initiate and maintain sleep. Wood et al. (2009) found that gratitude may help reduce sleep-onset latency and facilitate sleep efficiency by altering sleep-related cognitive processes. Although gratitude can, to some extent, boost subjective sleep quality, more research on its clinical efficacy and potential mechanisms are needed (Alkozei et al., 2019).

### 4.4. Subjective stress

Gratitude can also relieve psychological stress by decreasing negative emotions and fostering positive thoughts, emotions, and behaviors, thus affecting the development of CVD (O’Connell and Killeen-Byrt, 2018). Thrombosis and abnormalities in hemostasis, including increased platelet aggregability, have been found in patients with chronic stress (Levine et al., 1985; Räikkönen et al., 1996). Studies have shown that gratitude has a significant negative correlation with subjective stress levels (Wood et al., 2007) and can improve subjective or objective indicators of stress across time (Fredrickson, 2004; Wood et al., 2008). An extensive volume of evidence supports the potential of gratitude interventions in decreasing negative feelings and reducing their adverse effects on health (O’Connell et al., 2017; Wolfe and Patterson, 2017; Yang et al., 2018; Salces-Cubero et al., 2019). Furthermore, the results of several studies have demonstrated that various interventions to improve gratitude were also effective in reducing depression levels (Ramírez et al., 2014; Cheng et al., 2015; Watkins et al., 2015; Jackowska et al., 2016; Disabato et al., 2017; O’Connell et al., 2017; Sirois and Wood, 2017; Southwell and Gould, 2017; Wolfe and Patterson, 2017; Salces-Cubero et al., 2019).

**Figure 2:**
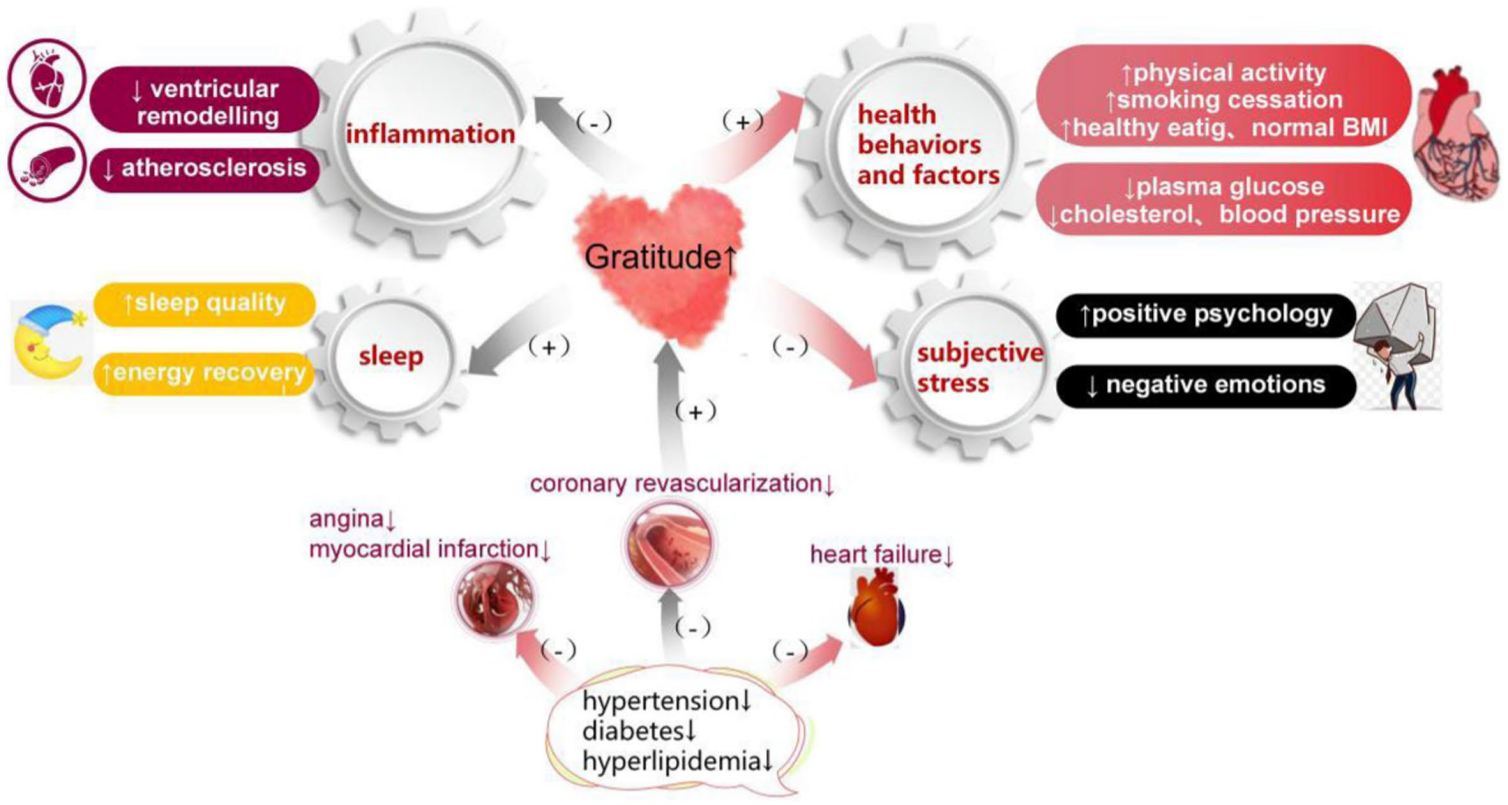
The relationship between gratitude and cardiovascular disease.

While gratitude can help patients with CVD, it can also be affected by CVD. As a positive psychological state, gratitude is influenced by many factors, such as physical health conditions, emotion regulation abilities, social support, subjective well-being (Salces-Cubero et al., 2019), psychological resilience, anxiety, and depression. Therefore, the severity of CVD can have crucial impact on gratitude. Poor physical condition is a risk factor for CVD and also affects gratitude levels, thus creating a vicious cycle in which poor health leads to increased CVD risk and lower gratitude levels, further increasing the risk of behaviors that induce poor health (Arnett et al., 2019). Studies have suggested that CVD, which can manifest as angina, myocardial infarction, HF, and coronary revascularization, can lower gratitude levels and cause both acute and chronic negative mental changes (Januzzi et al., 2000; Thombs et al., 2006; Lichtman et al., 2008; Smolderen et al., 2017). Hence, preventing and treating CVD can help improve gratitude levels to a large extent. Improvements in cardiovascular health can help promote positive mental states, which can build positive resources that foster well-being and enhance positive behaviors (Benjamin et al., 2019; Figure 2).

## 5. Mechanisms for the effect of gratitude on cardiovascular diseases

### 5.1. Inflammation

The levels of inflammatory factors, including interleukin-6 (IL-6), high-sensitivity C-reactive protein (hsCRP), soluble tumor necrosis factor receptor 1 (sTNFr1), interferon-gamma (IFN-γ), growth stimulation expressed gene 2 (ST2), and tumor necrosis factor-alpha (TNF-α) decreased among individuals with higher gratitude levels (Mills et al., 2015; Redwine et al., 2016; Huffman et al., 2016b; Hartanto et al., 2019). As part of the “neural alarm system,” the vCA1–amygdala pathway is crucial in the process of triggering inflammation (Irwin and Cole, 2011; Eisenberger and Cole, 2012). Gratitude may reduce inflammatory processes by increasing support giving and favoring decreased amygdala activity (Hazlett et al., 2021). Inflammation, a recognized risk factor for both atherosclerosis progression and plaque rupture, can directly accelerate atherosclerosis progression (Levine et al., 2021). Several studies have suggested that elevated hsCRP levels after ACS are independently associated with an increased risk of mortality (Zebrack et al., 2002; James et al., 2003; Ribeiro et al., 2014). In a prospective observational study of patients with ACS, Huffman et al. (2016a) reported a correlation between decreased levels of TNF-α and increased gratitude. Further, chronic pro-inflammatory factors can cause endothelial cell dysfunction by inducing chronic inflammatory processes. Some studies on vascular endothelial function have found that gratitude is associated with improvement in vascular endothelial function. For example, in a cohort study of patients with ACS, Celano et al. (2017) found that gratitude is associated with reduced markers of endothelial dysfunction, including endothelin-1 (ET-1) and soluble intercellular adhesion molecule-1 (sICAM-1).

Inflammation has also been implicated in the pathogenesis and prognosis of HF (Bouras et al., 2014). Pro-inflammatory factors are activated in asymptomatic HF, and their increase leads to worsening congestive HF (Bozkurt et al., 2010). In a randomized controlled trial among patients with asymptomatic HF, multiple inflammatory biomarkers, such as CRP, TNF-α, IL-6, and sTNFr1, were extracted from the patient’s blood before and after an 8-week gratitude diary intervention. At the end of the study, the biomarkers were checked again and were lower in the gratitude intervention group than in the usual treatment group (Redwine et al., 2016). Similar findings were reported in another HF study (Mills et al., 2015). In a study of 1,054 adults, Hartanto found that decreases in personal gratitude were significantly associated with decreases in IL-6 (Hartanto et al., 2019). This suggests that gratitude may suppress atherosclerosis and inhibit ventricular remodeling by reducing inflammation, which reduces the risk of major adverse cardiovascular events.

### 5.2. Autonomic nervous system

Gratitude may influence the occurrence and development of various CVD by increasing parasympathetic tone and decreasing heart rate and diastolic blood pressure. In a study of 32 healthy volunteers, the results showed that the average heart rate during a gratitude intervention was lower than during resentment intervention (Kyeong et al., 2017). A randomized blinded trial by Jackowska et al. (2016) and Redwine et al. (2016) found that gratitude interventions were associated with a decrease in diastolic blood pressure compared with no treatment controls. The limbic system in the human brain, especially the amygdala, is the neural basis of emotion processing. Increased amygdala activation can trigger strong emotional reactions including autonomic and endocrine responses. Gratitude levels are related to activity in the medial prefrontal cortex. Some evidence suggests that gratitude interventions may decrease sympathetic nervous system activity by reducing amygdala reactivity and activating the ventral striatum and diaphragm area (Hazlett et al., 2021). However, increased sympathetic tone, an important predictor of HF, is associated with higher mortality in patients with myocardial infarction (La Rovere et al., 1998; Machado et al., 2006; Shah et al., 2013). Increase in heart rate is associated with greater *sympathetic* than *parasympathetic* activity. Increased heart rate (>180 beats/min) was shown to shorten diastole duration (cardiac relaxation), further reducing coronary flow and cardiac output, increasing myocardial oxygen consumption, and aggravating myocardial ischemia and hypoxia.

### 5.3. Sympatho adrenomedullary system and renin–angiotensin–aldosterone system

Gratitude can inhibit the sympatho adrenomedullary (SAM) system and the renin–angiotensin–aldosterone system (RAAS), reducing cardiovascular responses to acute stress. Impaired baroreflex is regarded as a sensitive marker of autonomic cardiovascular regulation. Stress influences ischemic heart disease (Frasure-Smith and Prince, 1989; Frasure-Smith, 1991; Frasure-Smith et al., 1992, 1997) and has been demonstrated to cause myocardial ischemia in patients with coronary heart disease through several mechanisms (Krantz et al., 1991; Yeung et al., 1991; Manuck et al., 1992; Dakak et al., 1995). Studies have reported impaired vasomotor regulation of atherosclerotic epicardial coronary vasculature (Yeung et al., 1991) and coronary microcirculation (Dakak et al., 1995). The mu-opioid receptor, which is part of the opioid system, appears to be involved in stress relief and restoration of homeostasis. The experience of gratitude is accompanied by mu-opioid signaling, which explains the positive effects of gratitude on human physiological health. Self-reported levels of gratitude correlate with brain regions associated with emotional activities. Practicing gratitude can alleviate physiological stress and strain on the body and thus favorably influences health outcomes (Henning et al., 2017). In a study of patients with ACS, gratitude interventions reduced stress levels after ACS (Millstein et al., 2016). A double-blind randomized controlled trial included three groups: gratitude diary intervention group, bothered diary intervention group, and no intervention group. Individuals who received the gratitude diary intervention reported a decrease in self-reported depressive symptoms and stress (Cheng et al., 2015). In the study conducted by Rash et al., gratitude practices reduced stress and increased cardiac coherence, represented by a steady and ordered sinusoidal pattern in the heart rate variability waveform compared to baseline measurements. Subjecting participants to gratitude reflection in a dedicated laboratory can result in more ordered electrocardiogram waveforms and greater physiological coordination (Rash et al., 2011).

### 5.4. Hypothalamic–pituitary–adrenal axis

**Figure 3:**
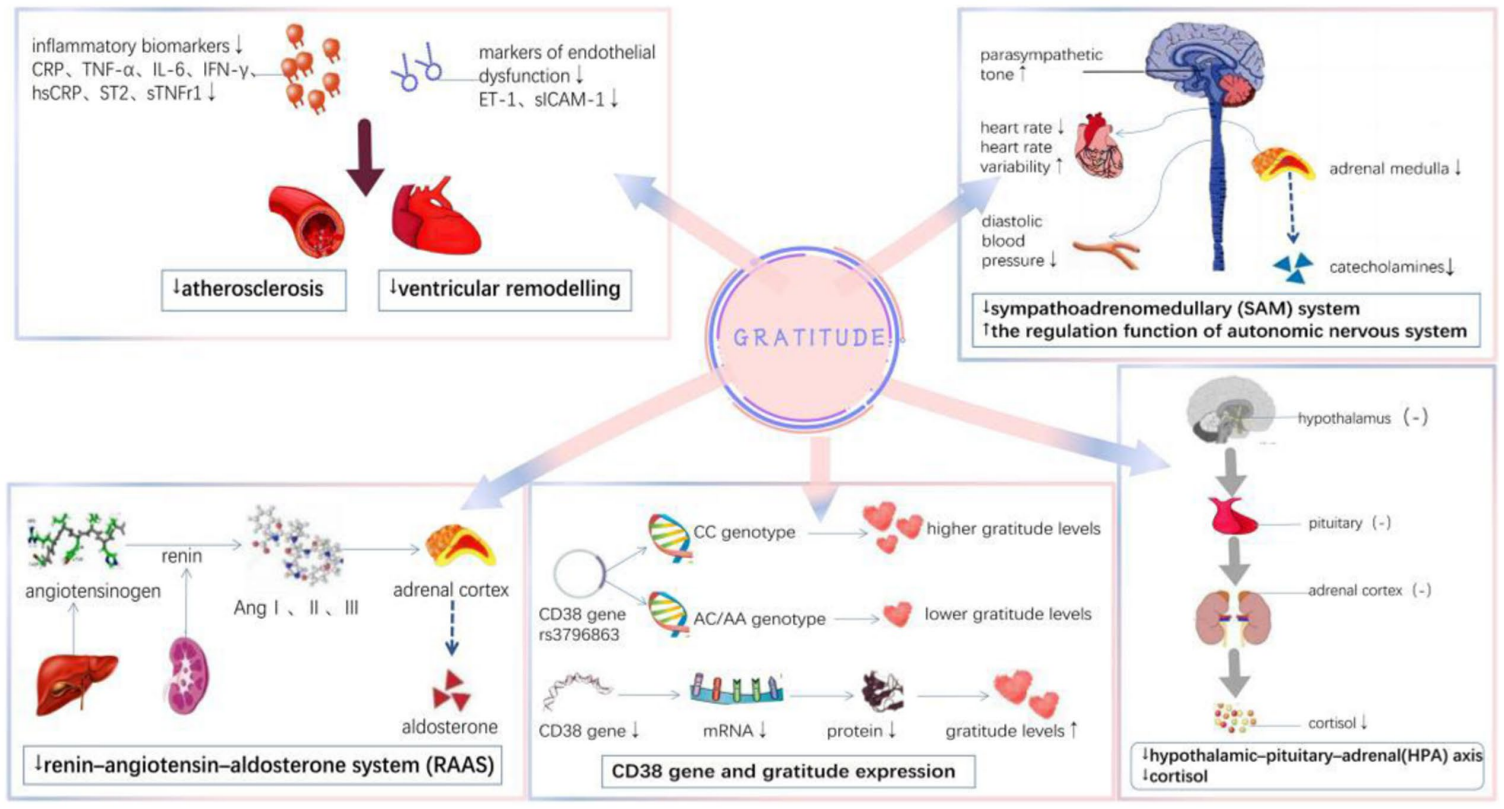
Mechanisms underlying the effect of gratitude on cardiovascular diseases.

Gratitude can inhibit the hypothalamic–pituitary–adrenal (HPA) axis and lower cortisol levels. Cortisol, considered a critical mediator of mental states and health-related outcomes, is the hormonal end-product of the HPA axis and can modulate neuroendocrine stress responses (Matvienko-Sikar and Dockray, 2017). The relationship between cortisol and mental state has been intensively investigated for many years. Stress has been frequently linked to elevated concentrations of endogenous cortisol, a hormone involved in the stress response (Biondi and Picardi, 1999). Writing gratitude journals can relieve pressure experienced during pregnancy, for example, and cortisol release is decreased as the pressure is relieved. In a randomized controlled trial of 46 pregnant women, gratitude interventions were provided 4 times per week for 3 weeks. The gratitude intervention group showed lower cortisol and stress levels in both wakefulness and sleep during pregnancy than the control group that received conventional therapy (Matvienko-Sikar and Dockray, 2017). Furthermore, CD38 gene expression can influence oxytocin signaling, and the oxytocin system has been implicated in the quality and frequency of gratitude expression. Studies have indicated that higher gratitude expression is related to lower CD38 expression, and functional genetic variation in CD38 is related to the quality and quantity of gratitude behavior (Algoe and Way, 2014; Figure 3).

## 6. Conclusion

In summary, gratitude therapy helps prevent the occurrence and development of CVD via changes in inflammation and the functions of the ANS, SAM system, RAAS, and HPA axis. Gratitude interventions have been shown to improve the physiological, psychological, and social functions of patients with CVD. However, the mechanisms by which gratitude impacts the health outcomes of patients with CVD are complex and require further research. There is a wide range of studies on the relationship between gratitude and variables related to social support, depression, well-being, sleep, and pain. However, these results could be strongly influenced by confounding factors, with the possibility of being exaggerated or diminished. Therefore, when exploring the mechanisms of gratitude on health-related variables in patients with CVD, confounding factors should be more strictly controlled. Future studies should focus on the construction of gratitude models that are applicable to various types of CVD. They could provide novel insights into the construction of health management models for patients with CVD.

## Data availability statement

The original contributions presented in the study are included in the article/supplementary material, further inquiries can be directed to the corresponding author.

## Author contributions

XW and CS developed the research question and search strategy, assessed publications for inclusion, and data synthesis and analysis. CS gave advice on publications for inclusion, and reviewed the manuscript. All authors participated in writing and reviewing the manuscript, contributed to the article, and approved the submitted version.

## Conflict of interest

The authors declare that the research was conducted in the absence of any commercial or financial relationships that could be construed as a potential conflict of interest.

## Publisher’s note

All claims expressed in this article are solely those of the authors and do not necessarily represent those of their affiliated organizations, or those of the publisher, the editors and the reviewers. Any product that may be evaluated in this article, or claim that may be made by its manufacturer, is not guaranteed or endorsed by the publisher.
